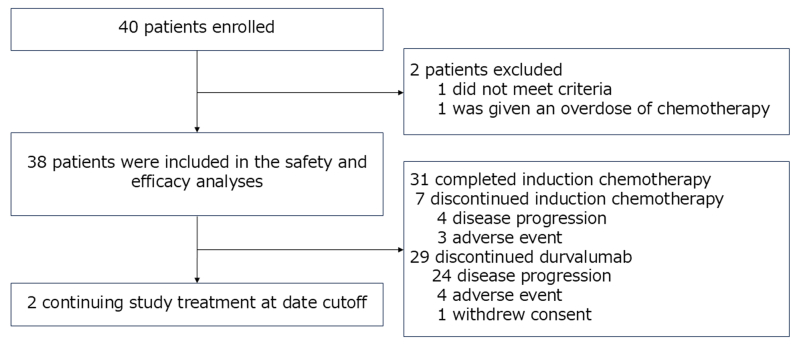# Turtle Study: A Phase 2 Study of Durvalumab Plus Carboplatin and Etoposide in Elderly Patients With Extensive-Stage SCLC (LOGiK 2003)

**DOI:** 10.1016/j.jtocrr.2025.100836

**Published:** 2025-04-21

**Authors:** Hidenobu Ishii, Koichi Azuma, Yuta Yamanaka, Hiroshige Yoshioka, Yukihiro Toi, Naoki Shingu, Katsuhiko Naoki, Masaki Okamoto, Yuko Tsuchiya-Kawano, Taishi Harada, Hiroyuki Inoue, Hiroshi Ishii, Kazunori Tobino, Chiho Nakashima, Yoshifusa Koreeda, Yasushi Hisamatsu, Shinsuke Tsumura, Takashi Inagaki, Keiko Mizuno, Takayuki Shimose, Isamu Okamoto

**Affiliations:** a Division of Respirology, Neurology, and Rheumatology, Department of Internal Medicine, Kurume University School of Medicine, Fukuoka, Japan; b Department of Thoracic Oncology, Kansai Medical University Hospital, Hirakata, Japan; c Department of Pulmonary Medicine, Sendai Kousei Hospital, Sendai, Japan; d Division of Respiratory Medicine, Saiseikai Kumamoto Hospital, Kumamoto, Japan; e Department of Respiratory Medicine, Kitasato University School of Medicine, Sagamihara, Kanagawa, Japan; f Department of Respirology, National Hospital Organization Kyushu Medical Center, Fukuoka, Japan; g Department of Respiratory Medicine, Kitakyushu Municipal Medical Center, Kitakyushu, Japan; h Department of Respiratory Medicine, Japan Community Health Care Organization Kyushu Hospital, Kitakyushu, Japan; i Department of Respiratory Medicine, Fukuoka University Hospital, Fukuoka, Japan; j Department of Respiratory Medicine, Fukuoka University Chikushi Hospital, Fukuoka, Japan; k Department of Respiratory Medicine, Iizuka Hospital, Iizuka, Japan; l Division of Hematology, Respiratory Medicine and Oncology, Department of Internal Medicine, Faculty of Medicine, Saga University Hospital, Saga, Japan; m National Hospital Organization Minamikyushu Hospital, Kagoshima, Japan; n Department of Thoracic Medical Oncology, Oita Prefectural Hospital, Oita, Japan; o Department of Respiratory Medicine, Kumamoto Regional Medical Center, Kumamoto, Japan; p Department of Medical Oncology and Hematology, Faculty of Medicine, Oita University, Oita, Japan; q Department of Pulmonary Medicine, Graduate School of Medical and Dental Sciences, Kagoshima University, Kagoshima, Japan; r Clinical Research Support Center Kyushu, Fukuoka, Japan; s Department of Respiratory Medicine, Graduate School of Medical Sciences, Kyushu University, Fukuoka, Japan

**Keywords:** Small cell lung cancer, Durvalumab, Elderly, Immunotherapy

## Abstract

**Introduction:**

The combination of immune checkpoint inhibitors with chemotherapy is the standard treatment for extensive-stage (ES) SCLC. However, its safety for elderly patients is not fully validated. We evaluated the safety and efficacy of durvalumab plus carboplatin and etoposide in elderly patients with ES-SCLC.

**Methods:**

In this prospective, single-arm, multicenter, phase 2 clinical trial, patients with ES-SCLC aged above or equal to 75 years received chemotherapy with up to four cycles of durvalumab 1500 mg on day 1, carboplatin at a dose equivalent to an area under the curve of 5 on day 1, and etoposide 80 mg/m^2^ on days 1 to 3 every 3 weeks as induction therapy. Maintenance therapy with durvalumab 1500 mg was administered every 4 weeks until disease progression or unacceptable toxicity. The primary end point was safety, and key secondary end points were objective response rate, progression-free survival, overall survival, quality of life, and Geriatric Assessment.

**Results:**

Between August 2021 and February 2023, 40 patients were enrolled at 17 institutions and 38 were assessable for safety and efficacy. Grade 3 or higher adverse events occurred in 36 patients (94.6%). The most common adverse events were hematologic, including grade 3 or higher neutropenia (76.3%) and febrile neutropenia (15.8%). The objective response rate, median progression-free survival, and median overall survival were 89.5%, 5.4 months, and 16.1 months, respectively. No decrease in quality of life or functional assessment scores was observed after treatment.

**Conclusion:**

Durvalumab plus carboplatin and etoposide was tolerable and expected to be effective in elderly patients with ES-SCLC.

## Introduction

SCLC is a neuroendocrine carcinoma that exhibits rapid growth and early spread to distant sites.^1^ Approximately 70% of patients with SCLC have extensive-stage (ES) disease at diagnosis.^2^ Chemotherapy is the first-line treatment option for patients with ES-SCLC. Anti–programmed death-ligand 1 antibodies have been found to provide clinical benefit in combination with chemotherapy and have become the standard of care.^3^^,^^4^ The IMpower133 trial revealed that the addition of atezolizumab to platinum-containing combination chemotherapy increased overall survival (OS) and progression-free survival (PFS) in patients with ES-SCLC,^3^ and the CASPIAN trial revealed an increase in OS with the addition of durvalumab.^4^

The aging of patients with cancer is a global issue.^5^ In Japan, more than 60% of patients diagnosed with having advanced lung cancer are above 75 years of age.^6^ Elderly patients tend to be less tolerant of toxic treatments than younger patients, because they typically have more comorbidities and may have impaired organ function, even when laboratory values are in the reference range. Therefore, evidence obtained in a study population dominated by young people cannot be applied directly to elderly patients. Even though most patients with lung cancer are elderly, the elderly population is not well represented in general clinical trials. Among patients enrolled in clinical trials in the National Cancer Institute collaborative group, less than 25% are 65 to 74 years old and less than 10% are 75 years of age or older.^7^ The IMpower133 trial contained only 10% of patients above 75 years of age^3^; thus, the proportion of older people in that trial was low, as in other trials. The CASPIAN trial also provided insufficient data on elderly patients.^4^

Although chemotherapy with immune checkpoint inhibitors is expected to be useful in elderly patients with SCLC, the data are insufficient. Therefore, we designed this phase 2 clinical trial to evaluate the safety and efficacy of durvalumab plus carboplatin and etoposide in elderly patients with ES-SCLC.

## Materials and Methods

### Study Design and Eligibility

This was an open-label, multicenter, single-arm phase 2 study conducted at 30 institutions in Japan. It was conducted in accordance with the provisions of the Declaration of Helsinki and was approved by the central review board of Clinical Research Network Fukuoka (CRB7180004). Written informed consent was obtained from all patients before enrollment. The trial is registered with the Japan Registry of Clinical Trials (jRCTs071210050).

Patients were aged 75 years and older with treatment-naive ES-SCLC. The eligibility criteria were Eastern Cooperative Oncology Group performance status (PS) of 0 or 1, no serious tumor-related complications, and measurable lesions. The patients were also required to have adequate organ functions which were defined as follows: absolute neutrophil count above or equal to 1500/liter, platelet count above or equal to 100,000/liter, hemoglobin above or equal to 9.0 g/dL, aspartate and alanine aminotransferase less than or equal to 100 IU/liter, total bilirubin less than or equal to 1.5 mg/dL, and serum creatinine less than or equal to 1.5 mg/dL. Exclusion criteria included synchronous or metachronous (within a year) malignancies; active infection requiring systemic therapy; uncontrolled pleural effusion, pericardial effusion, or ascites requiring recurrent drainage procedures; requirement of systemic steroid therapy (≥10 mg/d of prednisolone); and interstitial pneumonia or pulmonary fibrosis.

### Study Procedures and Treatment

Patients received durvalumab 1500 mg on day 1, carboplatin at a dose equivalent to an area under the curve (AUC) of 5 on day 1, and etoposide 80 mg/m^2^ on days 1 to 3 every 3 weeks for up to four cycles as induction therapy. Subsequent maintenance therapy with durvalumab 1500 mg was administered every 4 weeks until disease progression or the development of unacceptable toxicity. Prophylactic cranial irradiation and chest irradiation after chemotherapy were not prescribed as protocol treatments, as they are not standard treatments in Japan.

Computed tomography scans for tumor assessment were carried out every 6 weeks for the first 24 weeks, every 8 weeks from week 25 to week 48, and thereafter every 12 weeks until disease progression. The Physical Well-Being (PWB) subscale score, Functional Well-Being (FWB) subscale score, Lung Cancer Subscale (LCS) score, and FACT-L TOI were tabulated using the FACT-L questionnaire^8^ before enrollment and at 6 and 12 weeks after the start of treatment. Instrumental activities of daily living (IADL)^9^ were measured at baseline and 6 weeks after treatment as a Geriatric Assessment.

### Study Outcomes

The primary end point was safety, as measured by adverse events according to the Common Terminology Criteria for Adverse Events version 5.0, laboratory analyses, vital signs, and physical examination. The secondary end points were objective response rate (ORR), median PFS, 12-month OS, completion rate for four cycles of induction chemotherapy with durvalumab plus carboplatin and etoposide, Functional Assessment of Cancer Therapy—Lung Scale score, and IADL score.

### Statistical Analysis

The primary objective of this study was to evaluate the safety of the protocol treatment in elderly patients with ES-SCLC. It was not designed to be confirmatory, with a predetermined statistical threshold. Therefore, a sample size of 40 patients was set as the target number of patients to be enrolled in a period of 18 months. The statistical power required to detect adverse events was evaluated for 35 patients, assuming five ineligible patients. Adverse events with an incidence rate of 5% or more were expected to be observed in one or more patients with a probability of 83.4%.

ORR was defined as the proportion of patients who achieved a complete response or a partial response according to the Response Evaluation Criteria in Solid Tumors (version 1.1). PFS was defined as the period from the start of induction therapy to the date of disease progression or death due to any cause. OS was measured from the initiation of treatment until the date of death or last follow-up.

## Results

### Patients and Characteristics

A total of 40 patients were enrolled at 17 institutions between August 30, 2021, and February 28, 2023. Among them, one did not meet the criteria and another one was given an overdose of chemotherapy (Supplementary Fig. 1). A total of 38 patients met the eligibility criteria and were included in the safety and efficacy analyses. Baseline patient characteristics are found in Table 1. The median age was 78 (range, 75–88) years. Furthermore, 32 patients (84.2%) were male, and 97.4% patients had a history of smoking. Two (5.3%) patients were diagnosed with having combined SCLC, and the remaining patients had pure SCLC. Among all patients, 31 (81.6%) received the planned maximum of four cycles of platinum–etoposide plus durvalumab. After four cycles of induction therapy, one patient had disease progression and one patient discontinued protocol treatment due to adverse events; thus, 29 patients received maintenance therapy.

**Table 1:** Patient Characteristics

Characteristics	Patients
Age, y	
Median	78
Range	75–88
Sex, n (%)	
Male	32 (84.2)
Female	6 (15.8)
Smoking history, n (%)	
Never	1 (3.6)
Yes	37 (97.4)
Histology, n (%)	
Pure SCLC	36 (94.7)
Combined SCLC	2 (5.3)
ECOG PS, n (%)	
0	14 (36.8)
1	24 (63.2)

ECOG, Eastern Cooperative Oncology Group; PS, performance status.

### Safety

Median duration of follow-up was 11.3 (range: 2.7–27.8) months. Table 2 summarizes the all-grade adverse events. Adverse events of any cause and grade occurred in 38 (100%) of 38 patients, and grade 3 or higher adverse events occurred in 36 patients (94.6%). Grade 4 leucopenia and neutropenia occurred in seven patients (18.4%) and 20 patients (52.6%), respectively. Febrile neutropenia was observed in six patients (15.8%). Although four patients (10.5%) developed grade 3 thrombocytopenia, there was no grade 4 thrombocytopenia. Deaths due to adverse events of any cause occurred in two patients (5.3%) (one from lung infection and one from pneumonitis).

**Table 2:** Treatment-Related Adverse Events

Adverse Event	Any Grade			Grade 3+4		
	n	%	95% CI	n	%	95% CI
Leukopenia	32	84.2	68.7–94.0	23	60.5	43.4–76.0
Neutropenia	34	89.5	75.2–97.1	29	76.3	59.8–88.6
Anemia	38	100	90.7–100.0	8	21.1	9.6–37.3
Thrombocytopenia	33	86.8	71.9–95.6	4	10.5	2.9–24.8
Febrile neutropenia	6	15.8	6.0–31.3	6	15.8	6.0–31.3
Hypoalbuminemia	35	92.1	78.6–98.3	2	5.3	0.6–17.7
ALT increased	15	39.5	24.0–56.6	2	5.3	0.6–17.7
Serum amylase increased	16	42.1	26.3–59.2	2	5.3	0.6–17.7
Hyponatremia	32	84.2	68.7–94.0	2	5.3	0.6–17.7
Hypokalemia	8	21.1	9.6–37.3	3	7.9	1.7–21.4
Anorexia	19	50	33.4–66.6	3	7.9	1.7–21.4
Malaise	26	68.4	51.3–82.5	3	7.9	1.7–21.4
Enterocolitis infections	2	5.3	0.6–17.7	2	5.3	0.6–17.7
Lung infection	3	7.9	1.7–21.4	3	7.9	1.7–21.4
Rash	4	10.5	2.9–24.8	0	0	0.0–9.3
Colitis	1	2.6	0.1–13.8	1	2.6	0.1–13.8
Diarrhea	7	18.4	7.7–34.3	2	5.3	0.6–17.7
Pneumonitis	6	15.8	6.0–31.3	2	5.3	0.6–17.7
Hyperthyroidism	1	2.6	0.1–13.8	0	0	0.0–9.3
Hypothyroidism	1	2.6	0.1–13.8	0	0	0.0–9.3
Hyperglycemia	1	2.6	0.1–13.8	1	2.6	0.1–13.8

*Note:* Treatment-related AEs of grade 3 or 4 that occurred in more than 5% of patients or any immune-related AEs.

AE, adverse event; CI, confidence interval; ALT, alanine transaminase.

### Dose Reduction and Discontinuation

The dose was reduced by one level in the event of grade 4 neutropenia, grade 4 thrombocytopenia, more than or equal to 3 grade febrile neutropenia, or more than or equal to grade 3 nonhematologic toxicity (Supplementary Table 1). The dose reduction status of chemotherapy at each cycle of induction chemotherapy is found in Supplementary Table 2. Adverse events that required dose reduction were 13 cases of neutropenia, three cases of febrile neutropenia, two cases of both neutropenia and febrile neutropenia, and one case each of diarrhea, fever, and fatigue. Adverse events leading to discontinuation were reported in seven patients (18.4%). The reason for discontinuation was pneumonitis in three cases, colitis in one case, and PS decline in three cases.

### Efficacy

**Figure 1:**
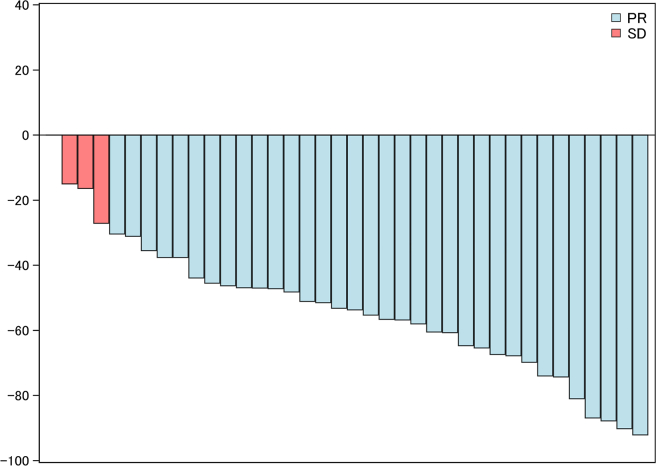
Best percent change in target tumor burden from baseline. PR, partial response; SD, stable disease.

**Figure 2:**
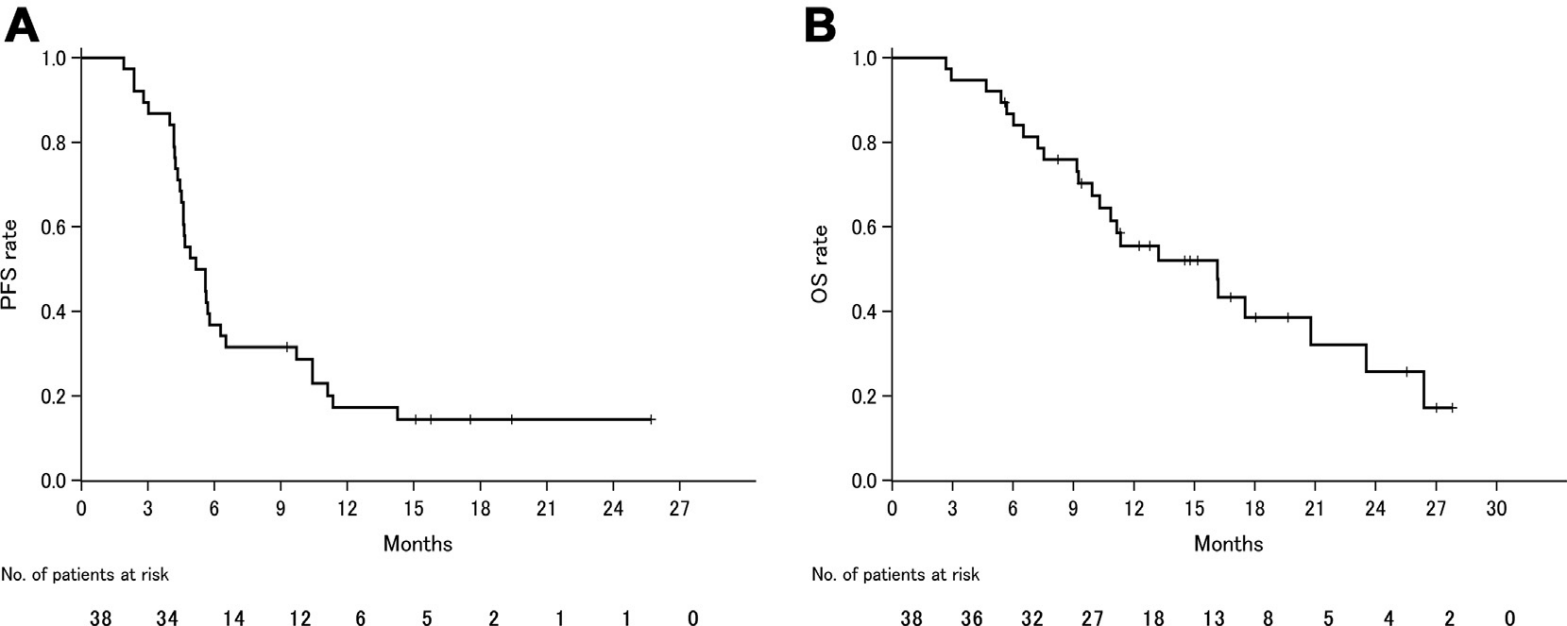
Kaplan-Meier survival curves for (*A*) PFS and (*B*) OS. OS, overall survival; PFS, progression-free survival.

The ORR was 89.5% (95% confidence interval [CI]: 75.2%–97.1%), with 34 patients achieving a partial response (Table 3 and Fig. 1). At the time of data cutoff, a total of 32 PFS events had occurred, for a median PFS of 5.4 months (95% CI: 4.6–6.3 mo; Fig. 2*A*). The 6-month and 12-month PFS rates were 36.8% (95% CI: 22.4%–52.6%) and 17.2% (95% CI: 6.9%–31.0%), respectively. The median OS was 16.1 months (95% CI: 10.3–23.5 mo), and the 1-year OS rate was 55.5% (95% CI: 39.1%–71.3%; Fig. 2*B*).

**Table 3:** Summary of Efficacy

Result	Durvalumab + Carboplatin and Etoposide
Best overall response	
Complete response	0
Partial response	34 (89.5)
Stable disease	4 (10.5)
Progressive disease	0
Objective response rate, %	89.5
95% CI	75.2–97.1
Median PFS, mo	5.4
95% CI	4.6–6.3
6-mo PFS rate, %	36.8
95% CI	22.4–52.6
12-mo PFS rate, %	17.2
95% CI	6.9–31.0
Median OS, mo	16.1
95% CI	10.3–23.5
6-mo OS rate, %	86.8
95% CI	74.3–95.5
12-mo OS rate, %	55.5
95% CI	39.1–71.3

CI, confidence interval; OS, overall survival; PFS, progression-free survival.

### Patient-Reported Outcome Measures

**Figure 3:**
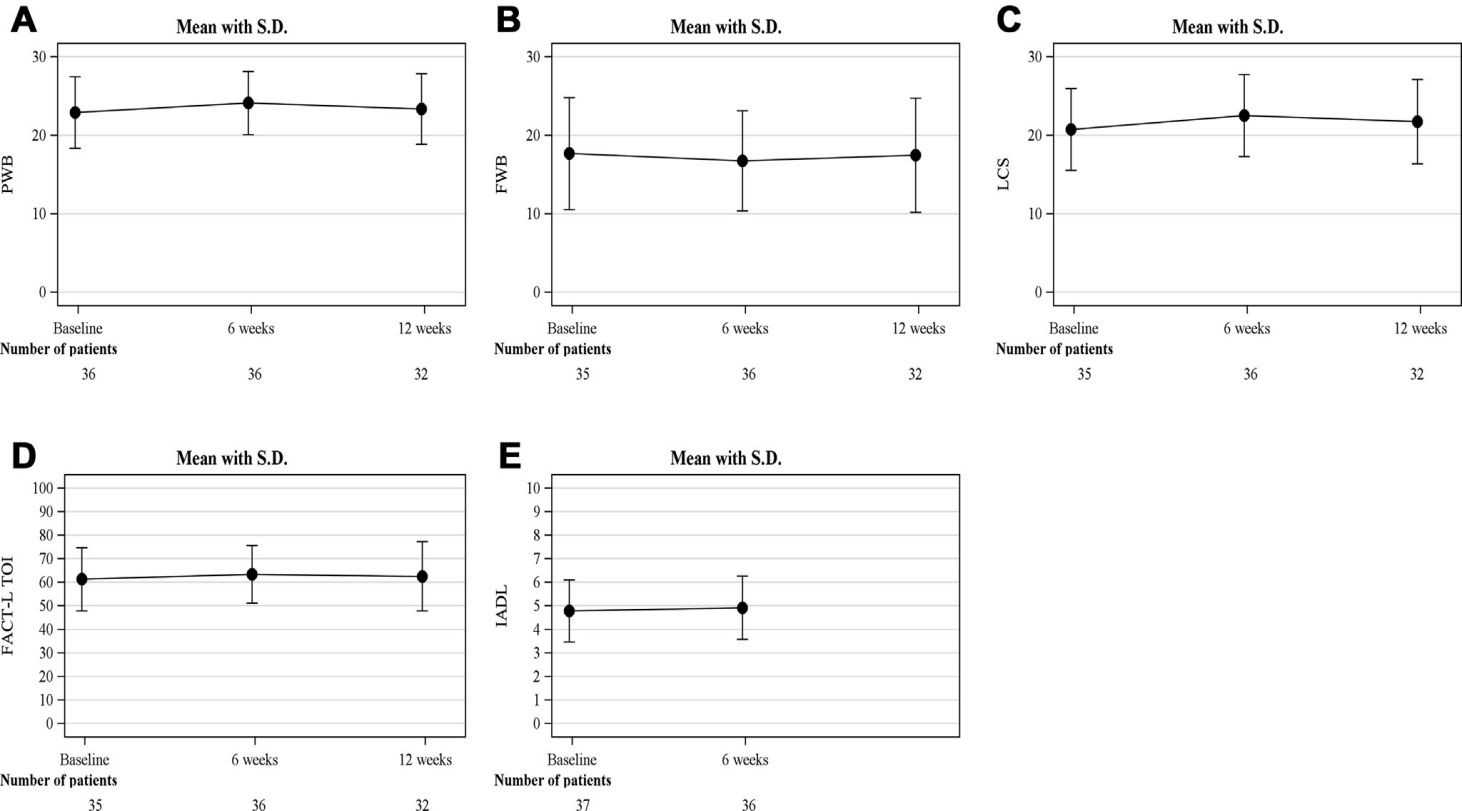
Change in (*A*) PWB; (*B*) FWB; (*C*) LCS, (*D*) FACT-L; and (*E*) IADL from before to after treatment. FWB, functional well-being; LCS, lung Cancer Subscale; PWB, Physical Well-Being.

There was no change in the PWB subscale score, FWB subscale score, LCS score, or FACT-L TOI, which is the sum of these, from before treatment to 6 and 12 weeks after treatment (Fig. 3*A*–*D*). There was also no change in IADL scores from before the start of treatment to 6 weeks after treatment (Fig. 3*E*).

## Discussion

To our knowledge, this is the first prospective phase 2 study of elderly patients with ES-SCLC. Treatment with carboplatin at a dose equivalent to an AUC of 5 plus etoposide 80 mg/m^2^ is often used for elderly patients with ES-SCLC in Japan, following the results of the randomized phase 3 JCOG 9702 trial.^10^ In this study, the carboplatin and etoposide doses used were according to those in JCOG9702 that were appropriate for the treatment of elderly patients with ES-SCLC.

In this study, 94.6% of patients had grade 3 or higher adverse events, compared with 62% in the CASPIAN study.^4^ The most frequent adverse events were mainly hematologic. In JCOG9702, the incidence of hematologic toxicity of grade 3 or higher was 54% for leukopenia, 95% for neutropenia, 56% for thrombocytopenia, and 29% for anemia.^10^ The incidence rates in this study were 76.0%, 88.6%, 24.8%, and 27.3%, respectively. There was no enhancement of hematologic toxicity with the addition of durvalumab to chemotherapy in elderly patients with ES-SCLC. The rate of treatment-related deaths was similar to that in the CASPIAN trial, suggesting that durvalumab plus carboplatin and etoposide therapy is relatively safe for elderly patients with ES-SCLC.

The response rate in the CASPIAN trial was 58% in the platinum combination chemotherapy group versus 68% in the durvalumab plus platinum combination chemotherapy group.^4^ In this study, a high response rate of 89.5% was observed. This is higher than the 73% response rate obtained with carboplatin plus etoposide therapy in the JCOG9720 trial and may be due to the add-on effect of durvalumab, as in the CASPIAN trial. PFS and OS were comparable to those of the CASPIAN trial. The 6- and 12-month PFS and 12-month OS were also comparable to those of the CASPIAN trial, suggesting that there is a long-term clinical benefit with durvalumab, even in elderly patients.

In recent years, it has been proposed that there is a need to perform not only oncological evaluations but also evaluations of functional status, physical ability and falls, comorbidities, depression, social activity/support, nutritional status, and cognitive function in elderly patients with cancer.^11^ We evaluated changes in quality of life (QOL) and Geriatric Assessment using the FACT-L and IADL as evaluation tools in patients before and after treatment. The FACT-L is a questionnaire consisting of 44 questions. Among them, PWB is a subscale that assesses physical satisfaction, FWB assesses functional satisfaction, and LCS is a subscale that assesses symptoms associated with lung cancer. These indices and the FACT-TOI, which is the sum of them, are used as indicators for assessing the QOL of patients with lung cancer. The IADL is a tool for assessing activities of daily living (ADL), and lower IADL scores have been found to be independently associated with early cessation of chemotherapy or poor chemotherapy tolerance. The results of this study suggest that chemotherapy with durvalumab plus carboplatin and etoposide for elderly patients with ES-SCLC does not impair patient QOL or ADL.

In recent years, it has been reported that aging causes a decline in immunity.^12^ This so-called immunosenescence is characterized by a decreased immune response to specific antigens and a tendency toward an increased inflammatory response, and it is associated with decreased susceptibility to infection and vaccine efficiency against pathogens and a sluggish inflammatory response.^12^ It has been suggested that immune checkpoint inhibitors may be less effective in the elderly due to immunosenescence.^13^ However, the results of this study suggest that immune checkpoint inhibitors are effective even in elderly patients.

This trial had several limitations. First, it was a single-arm study, and the sample size was relatively small. Second, we set PS 0 to 1 as an eligibility criterion. Most clinical trials are not conducted in the elderly population, but rather in a subset of the elderly population. The CASPIAN study provided no data in the elderly population; thus, it was not clear how effective this regimen would be in the elderly population. Consequently, it was necessary first to clarify the efficacy of this regimen in patients with good PS, and we decided to validate the efficacy of this regimen prospectively. We considered the study of tolerability in older patients with poor PS to be an issue for future study. Furthermore, the short observation period of this study may have resulted in an inadequate assessment of adverse events during the maintenance therapy period. However, there were only two cases in which maintenance therapy with durvalumab was continued at the end of the observation period, and the observation periods for these two cases were 15.1 and 19.6 months, respectively. Therefore, in most cases, adverse events were collected during the treatment period.

In conclusion, treatment with durvalumab plus carboplatin AUC 5 and etoposide 80 mg/m^2^ was tolerable and may be effective in elderly patients with ES-SCLC. As it can be administered without compromising QOL or ADL, this regimen could be a standard treatment for ES-SCLC, even in the elderly.

## CRediT Authorship Contribution Statement

**Hidenobu Ishii:** Conceptualization, Methodology, Data curation, Writing - original draft.

**Koichi Azuma:** Conceptualization, Methodology, Writing - original draft.

**Yuta Yamanaka:** Data curation, Writing - review & editing.

**Hiroshige Yoshioka:** Data curation, Writing - review & editing.

**Yukihiro Toi:** Data curation, Writing - review & editing.

**Naoki Shingu:** Data curation, Writing - review & editing.

**Katsuhiko Naoki:** Data curation, Writing - review & editing.

**Masaki Okamoto:** Data curation, Writing - review & editing.

**Yuko Tsuchiya-Kawano:** Data curation, Writing - review & editing.

**Taishi Harada:** Data curation, Writing - review & editing.

**Hiroyuki Inoue:** Data curation, Writing - review & editing.

**Hiroshi Ishii:** Data curation, Writing - review & editing.

**Kazuori Tobino:** Data curation, Writing - review & editing.

**Chiho Nakashima:** Data curation, Writing - review & editing.

**Yoshifusa Koreeda:** Data curation, Writing - review & editing.

**Yasushi Hisamatsu:** Data curation, Writing - review & editing.

**Shinsuke Tsumura:** Data curation, Writing - review & editing.

**Takashi Inagaki:** Data curation, Writing - review & editing.

**Keiko Mizuno:** Data curation, Writing - review & editing.

**Takayuki Shimose:** Formal analysis, Writing - review & editing.

**Isamu Okamoto:** Methodology, Writing - review & editing, Project administration.

## Disclosure

Dr. Hidenobu Ishii has received funding from AstraZeneca; honoraria from AstraZeneca, Chugai Pharmaceutical, KYORIN Pharmaceutical, and SYSMEX CORPORATION. Dr. Azuma has received honoraria from AstraZeneca, Merck Sharp & Dohme, Chugai Pharmaceutical, Ono Pharmaceutical, Bristol-Myers Squibb, and Takeda Pharmaceutical. Dr. Yoshioka has received research funding from Daiichi Sankyo, AstraZeneca, Janssen Pharmaceutical, Merck Sharp & Dohme, Novartis Pharma, Delta Fly Pharma, and Boehringer Ingelheim; consulting fee from Delta Fly Pharma; and honoraria from AstraZeneca, Boehringer Ingelheim, Taiho Pharmaceutical, Ono Pharmaceutical, Bristol-Myers Squibb, Novartis Pharma, Kyowa Kirin, Nippon Kayaku, Otsuka Pharmaceutical, Amgen, Pfizer, Nipro Pharma, Daiichi Sankyo, Merck Biopharma, Eli Lilly, Chugai Pharmaceutical, and Merck Sharp & Dohme. Dr. Toi has received honoraria from Ono Pharmaceutical, Bristol-Myers Squibb, Merck Sharp & Dohme, Chugai Pharmaceutical, and AstraZeneca. Dr. Shingu has received honoraria from AstraZeneca, Bristol-Myers Squibb, Takeda Pharmaceuticals, Chugai Pharmaceutical, and Merck Sharp & Dohme. Dr. Naoki has received research funding from Boehringer Ingelheim, Chugai Pharmaceutical Ono Pharmaceutical, Taiho Pharmaceutical, and Parexel International and honoraria from AstraZeneca and Chugai Pharmaceutical. Dr. Masaki Okamoto has received research funding, grant, and honoraria from Boehringer Ingelheim. Dr. Tsuchiya-Kawano has received honoraria from Bristol-Myers Squibb, Kyowa hakko Kirin, Taiho Pharmaceutical, Merck Sharp & Dohme, Chugai Pharmaceutical, Ono Pharmaceutical, AstraZeneca, and Takeda Pharmaceutical. Dr. Isamu Okamoto has received honoraria from Daiichi Sankyo, Eli Lilly, Chugai Pharma, Bristol-Myers Squibb, Merck Sharp & Dohme Oncology, AstraZeneca, Taiho Pharmaceutical, Ono Pharmaceutical, and Boehringer Ingelheim. The remaining authors declare no conflict of interest.

## Figures and Tables

**Supplementary Figure 1:**