# Supplementary material for: Turtle Study: A Phase 2 Study of Durvalumab Plus Carboplatin and Etoposide in Elderly Patients With Extensive-Stage SCLC (LOGiK 2003)
Source: JTO Clin Res Rep. 2025 Apr 21;6(7):100836. doi: 10.1016/j.jtocrr.2025.100836 (PMC12173610; doi:10.1016/j.jtocrr.2025.100836)
Supplement: Supplementary Tables 1 and 2 [file mmc1.docx]

Supplementary Table 1. Dose reduction level

|  | Carboplatin | Etoposide | Durvalumab |
| --- | --- | --- | --- |
| Level 0 | AUC 5 | 80 mg/m^2^ | 1500 mg/body |
| Level -1 | AUC 4 | 60 mg/m^2^ | Not administrered |
| Level -2 | AUC 3 | 40 mg/m^2^ |  |

Supplementary Table 2. Dose reduction status at each cycle

| Drug | Treatment cycle | 1 | 2 | 3 | 4 |
| --- | --- | --- | --- | --- | --- |
|  | Total patients | 38 | 37 | 35 | 32 |
| Carboplatin | AUC 5 | 38 (100%) | 18 (48.6%) | 17 (48.6%) | 15 (46.9%) |
|  | AUC 4 |  | 19 (51.4%) | 16 (45.7%) | 15 (46.9%) |
|  | AUC 3 |  |  | 2 (5.7%) | 2 (6.3%) |
| Etoposide | 80 mg/m^2^ | 38 (100%) | 19 (51.4%) | 18 (51.4%) | 16 (50.0%) |
|  | 60 mg/m^2^ |  | 18 (48.6%) | 16 (45.7%) | 15 (46.9%) |
|  | 40 mg/m^2^ |  |  | 1 (2.9%) | 1 (3.1%) |
